# Prevalence and Distribution of Segmentation Errors in Macular Ganglion Cell Analysis of Healthy Eyes Using Cirrus HD-OCT

**DOI:** 10.1371/journal.pone.0155319

**Published:** 2016-05-18

**Authors:** Rayan A. Alshareef, Sunila Dumpala, Shruthi Rapole, Manideepak Januwada, Abhilash Goud, Hari Kumar Peguda, Jay Chhablani

**Affiliations:** 1 Department of Ophthalmology, McGill University, Montreal, Quebec, Canada; 2 Smt. Kanuri Santhamma Retina Vitreous Centre, L. V. Prasad Eye Institute, Hyderabad, India; University of Massachusetts Medical School, UNITED STATES

## Abstract

**Purpose:**

To determine the frequency of different types of spectral domain optical coherence tomography (SD-OCT) scan artifacts and errors in ganglion cell algorithm (GCA) in healthy eyes.

**Methods:**

Infrared image, color-coded map and each of the 128 horizontal b-scans acquired in the macular ganglion cell-inner plexiform layer scans using the Cirrus HD-OCT (Carl Zeiss Meditec, Dublin, CA) macular cube 512 × 128 protocol in 30 healthy normal eyes were evaluated. The frequency and pattern of each artifact was determined. Deviation of the segmentation line was classified into mild (less than 10 microns), moderate (10–50 microns) and severe (more than 50 microns). Each deviation, if present, was noted as upward or downward deviation. Each artifact was further described as per location on the scan and zones in the total scan area.

**Results:**

A total of 1029 (26.8%) out of total 3840 scans had scan errors. The most common scan error was segmentation error (100%), followed by degraded images (6.70%), blink artifacts (0.09%) and out of register artifacts (3.3%). Misidentification of the inner retinal layers was most frequent (62%). Upward Deviation of the segmentation line (47.91%) and severe deviation (40.3%) were more often noted. Artifacts were mostly located in the central scan area (16.8%). The average number of scans with artifacts per eye was 34.3% and was not related to signal strength on Spearman correlation (p = 0.36).

**Conclusions:**

This study reveals that image artifacts and scan errors in SD-OCT GCA analysis are common and frequently involve segmentation errors. These errors may affect inner retinal thickness measurements in a clinically significant manner. Careful review of scans for artifacts is important when using this feature of SD-OCT device.

## Introduction

Quantitative assessment of the retina has become an important aspect of clinical management. Quantitative data is used to diagnose and guide treatment decisions in eyes with retinal diseases such as age related macular degeneration, diabetic macular edema and retinal vein occlusions. Thus, accurate in-vivo identification and characterization of retinal layers is essential. Several OCT scan modes are used to evaluate retinal pathology. The ganglion cell algorithm (GCA) is a recent technique that is used for inner retinal layer characterization, and is rapidly gaining popularity as a diagnostic tool in conditions involving the optic nerve and macula. The GCA algorithm software locates the outer boundary of the retinal nerve fiber layer (RNFL) and the outer boundary of the inner plexiform layer (IPL) and provides measurements of ganglion cell inner plexiform layer (GCIPL) thickness. An abundance of studies have shown that in-vivo quantitative measurement of retinal ganglion cell in the macula area can be an effective and powerful method for the evaluation of glaucomatous damage and progression.[1–8] Moreover, measurement of the thickness of the macular ganglion cell–inner plexiform layer thickness in various retinal diseases is now considered as a marker for retinal neurodegeneration.[9, 10] In addition, it appears to be a better biomarker of structural injury and can precisely measure the GCL+IPL thickness at baseline and assess changes occurring with time.[11]

To properly use the automated retinal layer segmentation, one must be aware of the limitations of such algorithms. Automated segmentation of distinct inner retinal layers has has been reported to correlate well with histology.[12] Lee et al and Oberwahrenbrock et al reported satisfactory reproducible measurements of GCIPL thickness using the Cirrus HD-OCT GCA algorithm.[13, 14]

Optical coherence imaging is challenging because of the complex interplay of myriad factors.[15] These factors include (a) software errors (misidentification of retinal layers, mirror artifact, cut edge artifact); (b) operator related error (degraded image scan, out of register artifact, off center artifact); and (c) patient related factors (motion artifact, off center artifact, degraded image scan, mirror artifact).[8, 16]These factors have been described for retinal thickness analyses. However, limited information is available regarding the artifacts and the errors of GCA.

The purpose of this study is to determine the frequency of artifacts, recognize the range of errors seen using the SD-OCT GCA algorithm, and to explain the causes of artifacts seen in healthy eyes.

## Methods

This was a retrospective observational study conducted at the L V Prasad Eye Institute, Hyderabad, India. The study was approved by the Institutional Review Board of the institute, and all methods adhered to the tenets of the Declaration of Helsinki. Written informed consent to participate was obtained from all subjects. Inclusion criteria were healthy eyes without any retinal or vitreoretinal interface abnormalities with good quality scan with signal strength of more than 6 and with refractive error between -6D and +3D. One eye of each patient was included in the study. Subjects were excluded if they had coexisting ocular diseases, uveitis, glaucoma, non glaucomatous optic neuropathy, history of intraocular surgery, myopia that was greater than -6D or hyperopia greater than +3 diopters or significant media opacities.

### OCT Image Acquisition and Processing

Spectral domain OCT scans were obtained by using the Cirrus® HD-OCT after pupillary dilation. The Macular Cube 512x128 scan protocol was used for all subjects. The protocol performs 128 B-scans and 512 A-scans per B-scan over 1024 samplings within a cube measuring 6 X 6 X 2 mm centered on the fovea. Images with signal strength < 6 were considered of poor quality and discarded.

**Fig 1:**
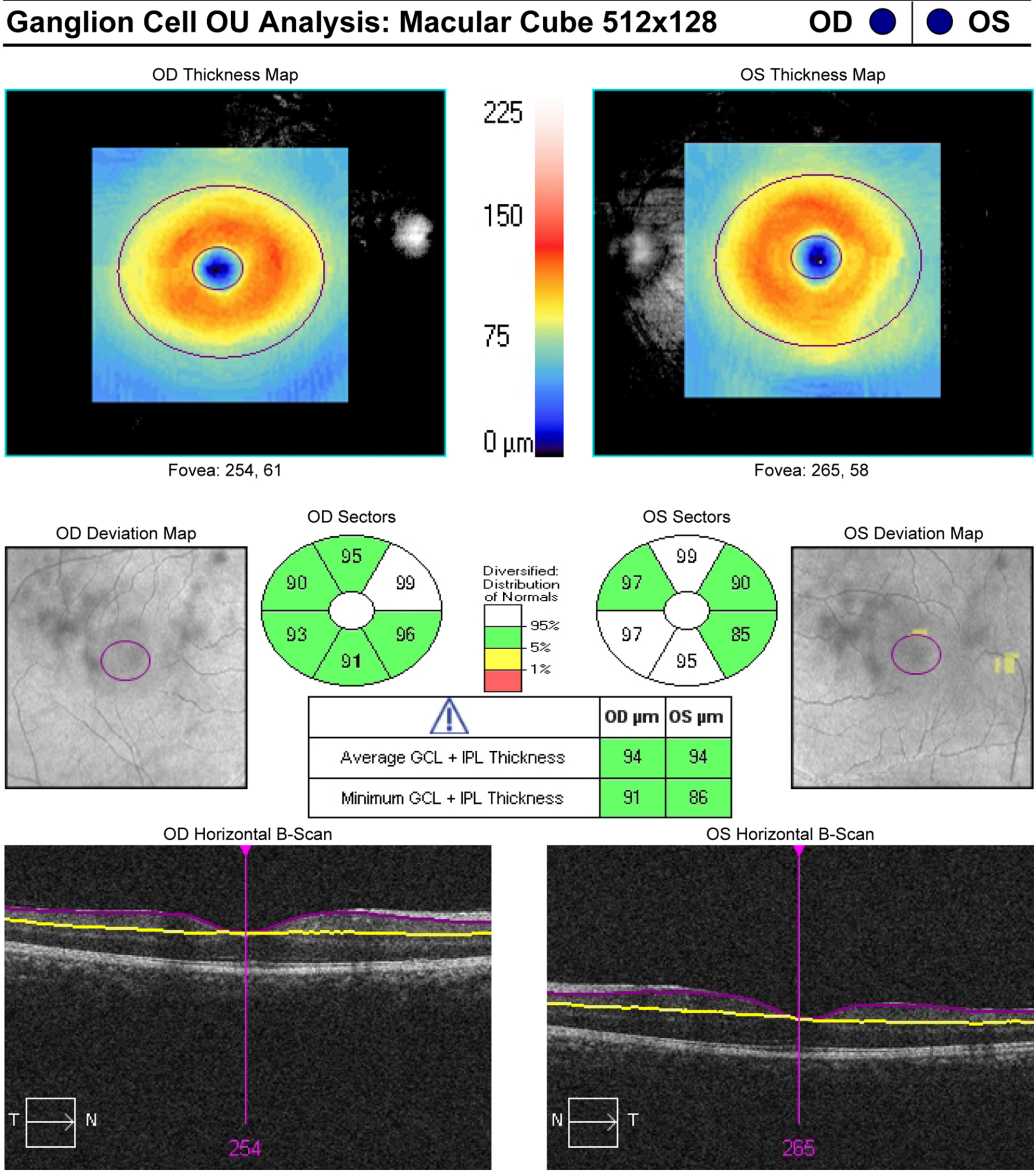
Example of macular ganglion cell inner plexiform layer (GCIPL) thickness measurement as determined automatically by optical coherence tomography: (Top maps) macular GCIPL thickness maps. Sectoral maps shows macular GCIPL thicknesses at superotemporal, superior, superonasal, inferonasal, inferior, and inferotemporal sectors. Sectoral thicknesses are measured in an elliptical annulus with a vertical outer radius of 2.0 mm and a horizontal radius of 2.4 mm. Deviation map shows the deviation of GCIPL measurements from age-matched healthy controls, shown as red (less 1% probability), yellow (1–5% probability), green (5–95% probability), and white (more than 95% probability). Cross-sectional scans at the level of fovea shows the segmentation of GCIPL. The outer border of the RNFL (retinal nerve fiber layer) is presented as a solid purple line and the outer border of the IPL is presented as a solid yellow line.

As described in our previous publications, the GCA algorithm was applied to the Macular Cube scans.[9, 10] The GCA algorithm identifies the outer boundary of the RNFL and presents it as a solid purple line and the outer boundary of the IPL and presents it as a solid yellow line, the difference between these two algorithm identified boundaries provides measurements of GCIPL thickness Fig 1.

The average, minimum (lowest GCIPL thickness over a single meridian crossing the annulus), and sectoral (superotemporal, superior, superonasal, inferonasal, inferior, inferotemporal) GCIPL thicknesses were measured in an elliptical annulus around the fovea (dimensions: vertical inner and outer radius of 0.5 mm and 2.0 mm, horizontal inner and outer radius of 0.6 and 2.4 mm, respectively). The GCA algorithm measures the mean GCIPL thickness for each sector, compares them to the internal normative database of the device, and generates a thickness map, a deviation map, and a color-coded significance map Fig 1. Measurements were displayed in green for normal range (P = 5%–95%), in yellow for borderline (1% < P < 5%), and in red for outside the normal range (P < 1%).

### OCT analysis

All 128 horizontal OCT B-scans acquired in the macular cube 512 × 128 protocol were examined by one evaluator (JC) for the presence of artifacts. Analysis of the images included infrared image, color-coded map and all individual 128 scans in an eye. Each scan was noted for segmentation error (inner or outer or both). Deviation of the segmentation line was classified into mild (less than 10 microns), moderate (10–50 microns) and severe (more than 50 microns). Each deviation, if present, was noted as upward or downward deviation. In addition to the evaluation of retinal “layers”, we investigated retinal “areas” where artifacts exist. Each artifact was further described as per location on the scan. Each scan was divided into central 1000 microns, nasal 2500 microns and temporal 2500 microns. Total scan area was divided in three zones: upper zone included 1^st^ to 41^st^ scan, central zone included 42^nd^ to 84^th^ scan and lower zone included 85^th^ to 128^th^ scan. Overall occurrence of the artifacts was described in these zones. Motion artifact was detected as misalignment of retinal vessels on rendered fundus infrared image.

### Statistical analysis

Descriptive statistics included mean and standard deviation. Statistical analyses were performed using commercial software (Stata data analysis and statistical software, version 12.1, StataCorp, College Station, TX). A p value of <0.05 was considered statistically significant. The number of segmentation errors in each OCT scan was analyzed according to different signal strengths to determine the relationship between signal strength and occurrence of errors using Spearman correlation.

## Results

The study comprised 30 eyes of 30 patients. Of these, 12 (40%) were female, and 18 (60%) were males. The mean patient age was 56.3±4.5 years. Of the total number of eyes observed, 13 (43.3%) were right and 17 (56.6%) were left eyes. Mean signal strength was 6.76 ± 0.81.

**Fig 2:**
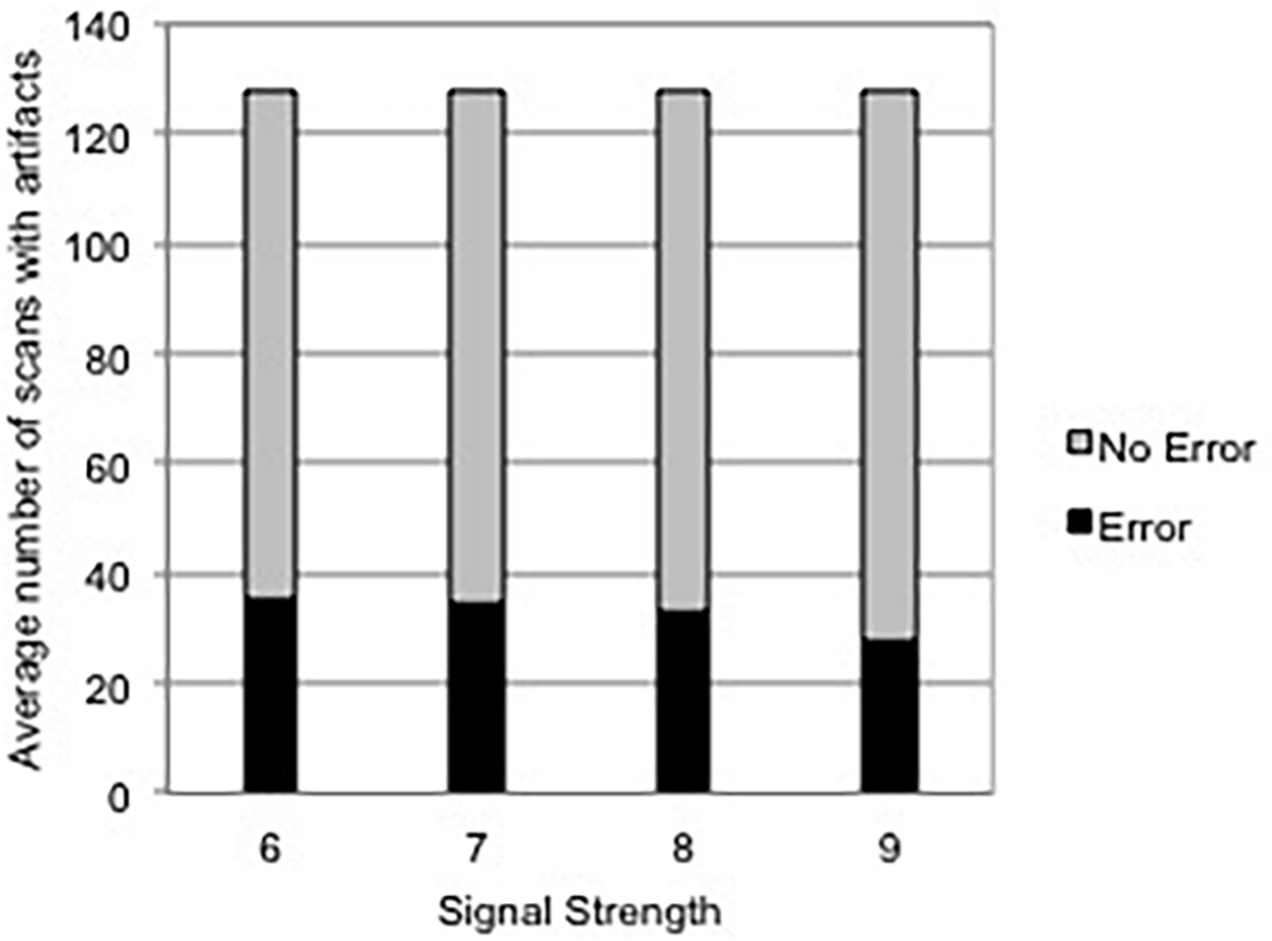
Histogram demonstrating average number of segmentation errors for each signal strength.

All 128 scans of each eye was analyzed. A total number of 3840 scans were examined. A total of 2811 (73.2%) scans were without any error, however, 1029 (26.8%) had scan errors. Mean number of scans in each eye was 35.84 (26%). Motion artifact on rendered fundus infrared image was noted in 16 (53.3%) eyes. The average number of scans with artifacts per eye was 34.3% and was not related to signal strength on spearman correlation (p = 0.36) Fig 2.

**Fig 3:**
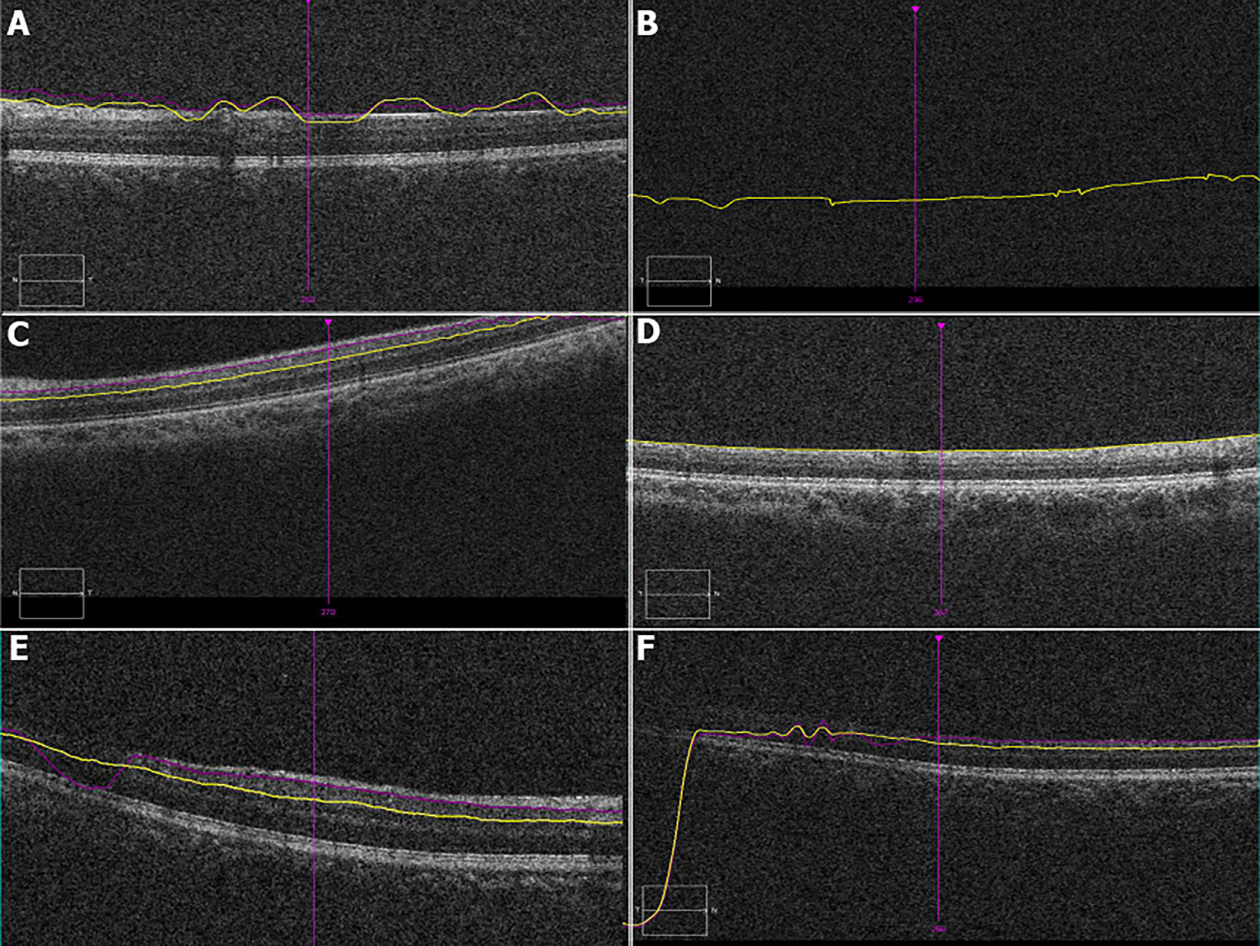
Composite figure demonstrating various artifacts on ganglion cell-inner plexiform layer automated segmentation: (A) Both, Inner and outer layer misidentification, note the purple does not follow the contour of the inner ganglion cell layer and the yellow line does not follow the outer inner plexiform layer; (B) Blink artifact: missing retinal image due to blink; (C) Out of register artifact: shifting of the scan superiorly with loss of information of temporal area; (D) Absence of outer segmentation line; (E) and (F) Degraded image with misidentification of layers in temporal (F) and nasal (E) regions. Of note, all signal strengths of these scans are >6.

Among the scans with errors (1029 scans), the most common type of error was segmentation error. Incomplete segmentation line error was in noted in the 128th scan of each eye 30/1029 (2.91%) and only affected the inner segmentation line (100%). Misidentification of the retinal layers most frequently involved the inner retinal layer (62%) compared to misidentification of the outer retinal layer 17.2%. Both layers were misidentified in 20.79% eyes. Other identified artifacts included degraded images (6.70%), blink artifacts (0.09%) and out of register artifacts (3.3%) Fig 3.

Upward Deviation of the segmentation line (47.91%) occurred more commonly then downward segmentation line deviation (33.4%). When comparing the degree of segmentation line deviation, severe deviation (40.3%) was more often noted then mild (38.6%) moderate (20.9%) deviation. When identifying each artifact as per horizontal location on the scan, artifacts were mostly located in the central scan area (16.8%). Overall 47%, 30% and 20% of artifacts existed in the lower, central and upper zones, respectively (Table 1).

**Table 1:** Percentage and characteristics of segmentation errors identified using the ganglion cell algorithm analysis^a^.

Type	Percentage
**Retinal layer misidentification**	
Inner	62%
Outer	17.2%
Both	20.79%
Missing Inner segmentation line	100%
Missing Outer segmentation line	0%
**Deviation**	
Upward	47.91%
Downward	33.43%
Both	18.65%
**Degree of deviation**	
Mild (less than 10 microns)	38.6%
Moderate (10–50 microns)	20.9%
Severe (more than 50 microns)	40.3%
**Affected Scan area**	
Nasal 2500 microns	11.2%
Central	16.8%
Temporal 2500 microns	11.4%
**Affected zones**	
Upper Zone (1^st^ - 41^st^ scan)	20%
Central Zone (42^nd^ - 84^th^ scan)	30%
Lower Zone (85^th^ -128^th^ scan)	47%

^a^ = Percentages are calculated out of total scans with errors (1029 scans), except for motion artifacts which is calculated from total number of eyes (30)

## Discussion

The clinical benefits of automated delineation of GCIPL are precise quantitative assessment of inner retinal layers and longitudinal assessment of inner retinal pathological processes. This may become a potentially valuable tool for the assessment of effects of novel therapeutic substances.[17–20] Of note, this has been used recently for patients with multiple sclerosis, where inner retinal layers have been shown to undergo thinning.[21–23] Therefore, accurate segmentation of layers without significant artifacts is necessary for better clinical decisions. Despite using the latest commercial retinal layer segmentation algorithms in this study, we noted that errors of retinal thickness measurement were present. The mean error rate using the GCA algorithm was (26.8%).

Our study identified several types of clinically important artifacts generated by SD-OCT GCA algorithm scanning, including those previously reported in SD OCT. Most artifacts observed with SD OCT in this study were related to software errors, rather than patient-associated or instrument operator errors. Most common algorithm software–related artifact was misidentification of the retinal boundaries especially inner retinal boundary. The most obvious cause of error was hyper-reflectivity of RNFL, which could result from poor signal quality of the SD-OCT image or outright failure of the segmentation algorithm. Operator related out-of–register artifact was noted in all 128 scans of one eye. Patient related blink artifact was noted only in a single scan.

Ray et al observed misidentification artifacts of the inner and outer retinal interfaces in eyes with macular disease using Stratus OCT when the inner or outer retinal later was disrupted or obscured by a macular hole or a choroidal neovascular membrane. Interestingly, when they evaluated scans of normal eyes they did not note inner and outer retina misidentification artifact and only observed artifact related to user error.^16^ In contrast to their findings, while evaluating the GCIPL of normal eyes with a clear interface, we noted misidentification artifacts involving the inner and outer retinal segmentation line present in one fourth of the volume scans. In our study, overall, misidentifications of the inner retinal layer (62%) were most common, followed by outer retina layer (17.2%). Misidentification of both layers was observed in 20.79% of scans.

Further analysis also revealed that vertical deviations were associated with GCIPL thickness changes. The present analysis suggests that the vast majority of these vertical deviations are of a severe (more than 50 microns) degree in 40.33% scans and moderate (10–50 microns) degree deviations were present in 29.9% eyes. In our study upward, downward deviations, or both were present in 47.9%, 33.4% and 18.6% eyes respectively. While analyzing our data, the distribution of the majority of artifacts was in the inferior zone, a possible explanation for this is patient fatigue. Similarly, we found the out of register artifact in the 128^th^ scan of all the eyes, which also could be due to fatigue. Severity of the deviation and affected zones would affect the average as well as sectoral GCIPL thicknesses. It has been reported that low OCT signal strength can induce segmentation errors.[24] In our report, however, the signal strength of the OCT scan was not associated in a significant manner with the existence of an error in segmentation (p = 0.36). This finding suggests that although the signal strength that is given in the OCT printout is not low, a segmentation error still can occur.

Using scan protocols with 128 B scans to acquire information about more peripheral areas may be problematic, as the more peripheral the subfield of interest, the more the software has to interpolate thicknesses. The macular GCIPL analysis printout of Cirrus HD-OCT results provides only one horizontal B-scan image centered at the fovea. As reported by Hwang et al, a simple way to survey a macular GCIPL segmentation error is by inspecting the macular GCIPL thickness map for (a) a blue to black funnel-like area (b) a pink to white area with a clear margin or (c) a generalized thinning of the macular GCIPL. They concluded that if one of these findings is noted, the possibility of a segmentation error should be studied. [25] While some investigators advise manual measurement of retinal thickness for patients in whom segmentation error that in more than one of the line scans,[26] the Cirrus® SD-OCT device does not allow correction of the segmentation error. Therefore, we could not evaluate the quantitative effect of artifacts on GCIPL measurements, nor were we able to manually measure the macular GCIPL thickness using the built in software. Other factors that may result in segmentation errors are pupil constriction and vitreous opacities. Dilating the pupil and asking the patient to move or blink they eye in an attempt to change the location of the opacity, might be helpful. [25] In general, if a segmentation error occurs in a healthy eye, we recommended that a repeat OCT examination is performed.

The Cirrus® SD-OCT device does not allow correction of the segmentation error. Therefore, we could not evaluate the quantitative effect of artifacts on GCIPL measurements. Artifacts in diseased eyes have not been evaluated in present study; therefore, we are unable to comment about GCA algorithm artifacts in diseased eyes. A study to elucidate the artifacts in diseased eyes is underway. To prevent the occurrence of artifacts, technicians from the manufacturer should calibrate the OCT device regularly. According to the manufacturer’s guidelines, only images with signal strengths of 6 or more should be considered for evaluation.

Finally, although GCA algorithm protocol on SD OCT remains a significant advance in the ability to image and assess the inner retina, scan artifacts marks a significant challenge to image quality and accuracy. In addition, errors in the detection of retinal layer boundaries and the measurement of retinal thickness are frequent with existing OCT analysis software. As we presented here, artifacts in SD OCT are not uncommon and clinicians must assess the ganglion thickness map because artifacts may impact OCT algorithm generated thickness measurements significantly. Clinicians should be careful whenever using these measurements in diagnostic or therapeutic decisions. The GCA analysis algorithm imaging remains a rapidly developing field, and continued revisions in software segmentation algorithms may provide increasingly reliable quantitative ganglion layer thickness data for improvement of not only understanding retinal disease pathology but also patient care.
